# Comparative Phenotype and Transcriptome Profiling in Some Grapevine Cultivars in Response to Drought Stress

**DOI:** 10.3390/plants15101464

**Published:** 2026-05-11

**Authors:** Igor Gavrilenko, Ekaterina Vodiasova, Victoria Uppe, Galina Maletich, Artem Pronozin, Yuri Plugatar, Sergey Dolgov, Pavel Khvatkov

**Affiliations:** 1 Federal State Funded Institution of Science, The Labor Red Banner Order Nikita Botanical Gardens—National Scientific Center of the RAS, Nikita, 298648 Yalta, Russia; igor.gavrilenko.95@bk.ru (I.G.); vikauppe@mail.ru (V.U.); maletich.galina@yandex.ru (G.M.); pronozinartem95@gmail.com (A.P.); plugatar.y@gmail.com (Y.P.); dolgov@bibch.ru (S.D.); khvatkov1987@gmail.com (P.K.); 2 A.O. Kovalevsky Institute of Biology of the Southern Seas of RAS, 299011 Sevastopol, Russia; 3 Institute of Cytology and Genetics, Siberian Branch of the Russian Academy of Sciences, 630090 Novosibirsk, Russia; 4 Branch of Shemyakin and Ovchinnikov Institute of Bioorganic Chemistry, 142290 Puschino, Russia

**Keywords:** drought tolerance, grapevine, mannitol, phenotyping, RNA-seq, *Vitis*

## Abstract

Drought is one of the main stress factors significantly affecting the growth, development and yield of agricultural crops. This study investigated the impact of drought stress on the grapevine. The 30 cultivars were classified as drought-tolerant, intermediately tolerant or sensitive. The phenotypic characteristics the number of new leaves, the number of second-order roots and the length of second-order roots (NL, NR2 and LR2 respectively) were identified as the most sensitive biometric characteristics. These parameters can be used to determine the optimal level of stress exposure for plants. Using transcriptomic data from five cultivars with different levels of tolerance, differentially expressed genes (DEGs) were identified in control plants and in plants under stress, as well as DEGs between different varieties when exposed to 2% mannitol. General patterns of gene expression under drought stress were subsequently identified, including the activation of antioxidant defense systems and changes in the metabolism and biosynthesis of glucan, cellulose, polysaccharides, monocarboxylic acids, fatty acids and metal transport and splicing processes. It is hypothesized that drought tolerance is determined by the increased expression of genes associated with glutathione metabolism and methylation processes.

## 1. Introduction

Grapevine global production reaches 70 million tons, occupying more than seven million hectares of land for harvesting [1,2]. As precipitation becomes more sporadic in these areas, grapevines are often affected by drought stress, which would seriously affect the yield and quality of grapevines and then restrict the development of the grapevine industry [3], impacting the economic sustainability of viticulture [4]. Grapevines generally exhibit an avoidance strategy in response to drought stress, maintaining stem water potential (Ψstem) above −1.5 MPa in non-irrigated conditions [5]. However, there is variability in drought responses between *Vitis* cultivars, and drought tolerant cultivars can serve as donors of drought resistance for the creation of elite, high-quality berry cultivars to improve their performance under drought [6]. Adaptation to climate change is an important step in the future of viticulture, which depends heavily on weather and climatic conditions [7]. Quite a few studies have looked at mechanisms by which rootstocks improve grapevine drought tolerance [8,9,10,11,12,13] and how plant–water relations are affected by rootstocks that maintain transpiration and root water uptake even to the point of risking hydraulic failure [9,14]. Yet, a better molecular mechanistic understanding of grapevine tolerance to drought is necessary to improve crop management and the development of new cultivars [15].

Several mechanisms have evolved to protect the plant from the adverse effect of drought stress [16]:

Several reactive oxygen species are generated during drought-induced oxidative stress, which are cytotoxic in nature. Plants have evolved complex antioxidant defense mechanisms, both enzymatic (superoxide dismutase (SOD), ascorbate peroxidase (APX), catalase (CAT), glutathione reductase (GR), etc.) and non-enzymatic (glutathione, alpha-tocopherol, flavonoids, carotenoids, ascorbate, etc. [17]), to defend against osmotic stress [18]. Studies have showed that differences can be observed in the activity of antioxidative enzymes and levels of their gene expression in tolerant and sensitive genotypes [19].

Plants adjust the osmotic pressure via the production of compatible solutes under salt and drought stress conditions. It includes sucrose, proline, quaternary ammonium compounds (hydroxyproline betaine, proline betaine, glycine betaine, etc.) and trehalose [20,21]. Proline plays main role in the protection of plants from osmotic stress and is one of the most studied plant compatible solutes [22]. The accumulation of proline in tissues helps in the detoxification of enormous ammonia, osmotic adjustments, membrane stabilization, scavenging of free radicals, shielding photosynthetic process, protecting mitochondrial functions and nitrogen and carbon reserves for growth and development after stress resistance [23].

Plants respond to drought stress by having anatomical, morphological and ultrastructure adaptations. Roots play a very important role in acclimating under stress. Adaptive characters for drought and salt stress tolerance include long roots, an intense root system and a high root density [24]. The thicker root structure absorbs greater amount of water when compared to thinner roots and roots in a high number can get in touch with greater water vapors available in soil [25].

The in vitro culture of plants on a medium with selection agents gives the chance to choose and regenerate plants with advantageous traits. The selecting agents generally utilized for in vitro drought screening include polyethylene glycol (PEG), mannitol, sorbitol and sucrose [16]. Sugar alcohols such as mannitol and sorbitol have often been used as metabolic inert osmotics in plant cell culture [26,27,28]. They can simply penetrate plasma membranes and cell walls resulting in an increase in osmotic pressure and lead to plasmolysis [19,28,29]. Mannitol has been often used to control the osmotic potential of the nutrient solutions in order to induce water-deficit conditions, especially in the root zone [30,31]. The influence of soil heterogeneity, environmental factors and climatic factors may create difficulties in screening at the field level. Therefore, the in vitro screening condition is considered to be beneficial over field screening [32]. Though the effect of drought stress cannot be determined only using in vitro screening, preliminary reports can predict the response of stresses. Further validation of response for stress can be done in the field conditions.

Moreover, in response to different stresses, plants are protected by complex regulatory networks in which transcription factors play a central role by regulating target gene expression leading to downstream physiological level changes [33]. The complexity of the processes involved in developing plant stress tolerance stems from the fact that the implementation of the genetic program is determined by a large number of external and internal factors. Various transcription factors (such as WRKY, MYB, ERF and the NAC transcription factor family) are involved in modulating the transcription of genes sensitive to abiotic stress.

Studies have also been conducted on grapevines to investigate the effects of drought on plant physiology and to identify the molecular mechanisms underlying drought tolerance. However, these studies have either compared two contrasting cultivars [34,35] or examined the effects of drought on specific tissues within a single cultivar [36,37,38,39,40]. These studies are limited in scope, as a comparative transcriptomic analysis of only two cultivars is unlikely to reveal patterns that are characteristic of all grapevines. The diversity of existing grapevine cultivars enables more comprehensive research into the effects of drought on cultivars with varying levels of resilience. However, no such studies have previously been conducted.

Thus, this study aimed to rank 30 grapevine cultivars according to their drought tolerance, based on changes in phenotypic and biochemical parameters. A comparative transcriptomic analysis was conducted on several cultivars with varying levels of drought tolerance to preliminarily identify common patterns and biological processes involved in the response to this type of stress across all cultivars. This approach will help to identify areas for further research into the mechanisms of grapevine drought tolerance.

## 2. Results

### 2.1. Clustering of Grapevine Cultivars According to Drought Resistance

**Figure 1:**
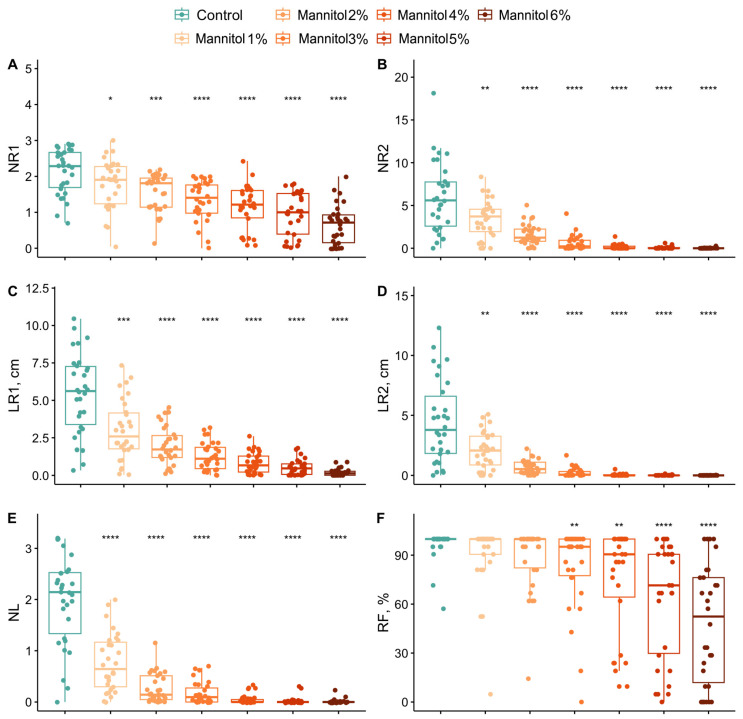
Biometric characteristics of each grapevine cultivar in relation to different concentrations of mannitol, with the number of first-order roots (NR1) (**A**), the number of second-order roots (NR2) (**B**), the length of first-order roots (LR1) (**C**), the length of second-order roots (LR2) (**D**), the number of new leaves (NL) (**E**) and the risogenesis efficiency (RF) (**F**) for thirty grapevine genotypes under control and drought-stress conditions. *, **, *** and **** indicate significance levels at *p* < 0.05, *p* < 0.01, *p* < 0.001 and *p* < 0.0001 according to the *t*-test, respectively. The results are presented with a boxplot (middle bar: median; box limits: upper and lower quartiles).

Nine phenotypic traits were examined in the presence of mannitol at concentrations ranging from 1% to 6%. The parameters studied were divided into two groups: biometric, the number of first-order roots (NR1), the length of first-order roots (LR1), the number of second-order roots (NR2), the length of second-order roots (LR2), the number of new leaves (NL) and the risogenesis efficiency (RF); and biochemical (chlorophyll a and b, proline and carotenoids). Changes in the biometric characteristics of each cultivar were analyzed in relation to different concentrations of mannitol (Figure 1). ANOVA revealed significant variations in biometric indicators across all levels of drought stress, when compared to the control group.

The number of first-order roots gradually decreased for all cultivars as the mannitol concentration increased, while the number of second-order roots dropped sharply by 2%. At mannitol concentrations above 3%, the number of second-order roots was almost zero for all cultivars. In contrast to NR1, the length of the first-order roots decreased more sharply with increasing stress levels, reaching close to zero for almost all cultivars at a mannitol concentration of 6%. Different levels of drought stress affected the length of second-order roots and the number of new leaves in the same way as the number of second-order roots; at a mannitol concentration above 3%, the number of second-order roots and new leaves became zero for almost all cultivars. It should be noted that risogenesis efficiency was the only biometric parameter that did not change significantly at low stress levels (mannitol concentrations of 1% and 2%). At this level of drought stress, risogenesis efficiency decreased for only a few cultivars, such as Akademik Avidzba, Malbec and Riesling. Most cultivars maintained sufficiently high risogenesis efficiency (>60%) even at a mannitol concentration of 5%. Genotypes such as Aligote, Ferkal, Kober 5BB, Livia and Syrah retained 100% risogenesis efficiency under maximum stress.

**Figure 2:**
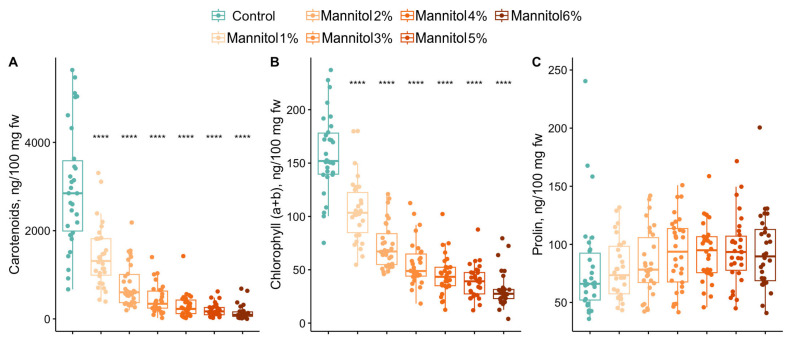
Biochemistry characteristics of each grapevine cultivar in relation to different concentrations of mannitol, with the contents of carotenoids (**A**), the contents of chlorophyll (a + b) (**B**) and the contents of proline (**C**) for thirty grapevine genotypes under control and drought-stress conditions. **** indicate significance levels at *p* < 0.0001 according to the *t*-test. The results are presented with a boxplot (middle bar: median; box limits: upper and lower quartiles).

A significant decrease in carotenoids and total chlorophyll was observed as stress levels increased compared to control growing conditions (Figure 2). At the same time, a sharp decrease in the carotenoid content occurred at the first levels of stress (1–2% mannitol), and at a mannitol concentration of more than 3%, the carotenoid content decreased for all cultivars. In the control groups, the carotenoid content ranged from 574.8 to 5932.6 ng/100 mg of fresh weight (fw), whereas at 3% mannitol, this range was 15.3–1441.3 ng/100 mg fw. Total chlorophyll a and b decreased steadily, reaching values of 0.3–86.1 ng/100 mg fw at a mannitol concentration of 6%. In control plants, this indicator ranged from 70.2 to 255.1 ng/100 mg fw. No significant changes in the proline concentration, an indicator of rapid stress, were detected 12 h after plant incubation began under stress conditions.

An analysis was conducted to determine the levels of drought stress experienced by different grapevine cultivars. This analysis showed that phenotypic parameters respond differently to mannitol concentrations (see Table 1, different letters in superscript indicate the significant drought effect.). The number of first-order roots is the only parameter that differs for each stress level (groups a–g). The length of the first-order roots varies across the stress levels up to 4% mannitol (groups a–e), but a further increase in the mannitol concentration does not lead to significant changes (groups e, ef and f). The number and length of second-order roots exhibit a consistent pattern across groups under control conditions and at 1%, 2% and 3% mannitol (groups a–d), with no significant differences observed at higher concentrations (group d). A decrease in the number of new leaves occurs at 1% (group b), then at 2–3% (group c) and at >4% (group d). The effectiveness of rhizogenesis gradually decreases and can only be separated into groups d and e at 5% and 6% mannitol, respectively. Stress levels of 1%, 2% and >3% mannitol can also be distinguished based on an analysis of carotenoid and chlorophyll a + b contents (groups a–c and a–d, respectively). Therefore, for all phenotypic parameters except proline, a mannitol concentration of 1% is stressful and causes a decrease in these parameters. At mannitol concentrations of more than 3%, no significant changes are observed and virtually all cultivars are in a state of maximum stress.

**Figure 3:**
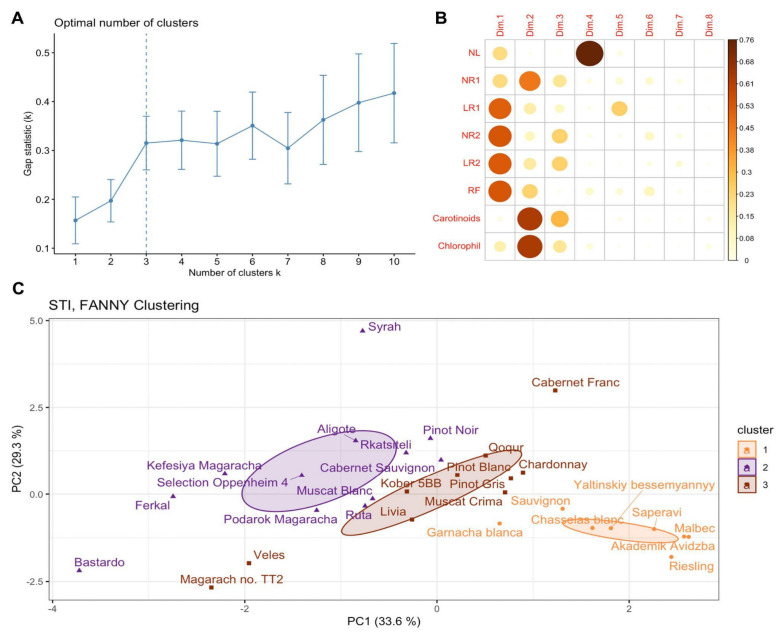
PCA and clustering of thirty grapevine cultivars according to the stress tolerance index (STI) to drought stress. (**A**) Gap statistic plot to determine the optimal number of clusters. (**B**) Contribution of the variables to the principal components. (**C**) Clustering of thirty grapevine cultivars. The color indicates the cluster group. The size of the concentration ellipses notes the normal probability (0.95).

A tolerance index (STI) was calculated for each cultivar and phenotypic trait: the higher the index, the more resistant the genotype (Table S2). This enabled us to cluster cultivars according to their drought-stress resistance. As no significant changes in proline were observed when the genotypes were exposed to mannitol, this parameter was excluded from the analysis. Therefore, PCA was only performed for the STI of eight phenotypic characteristics. Based on the contribution of STI values to the main components, the data were divided, with biochemical indicators (carotenoid and chlorophyll a + b content) contributing significantly to the second main component and biometric indicators (LR1, NR2, LR2 and RF) to the first (see Figure 3B). Cluster analysis of cultivars using the FANNY method revealed an optimal number of clusters k = 3 (Figure 3A). These clusters correspond to drought-sensitive genotypes (cluster 1), drought-resistant genotypes (cluster 2) and genotypes with intermediate resistance (cluster 3).

The spread of cultivars within the selected clusters varies. Sensitive cultivars form a more compact cluster, whereas resistant cultivars have a greater spread. This is because biometric and biochemical indicators react differently to stress. For instance, the Aligoté cultivar has an STI of 0.52 and 0.43 for carotenoid and chlorophyll a + b contents, respectively. Meanwhile, the biometric indicators NR1 and RF have an STI of 0.89 and 1.06, respectively. The STI for the remaining parameters (NL, LR1, NR2 and LR2) varies between 0.089 and 0.43, which is associated with a sharp decline when the mannitol concentration is increased to 4% (see Figure 1B,D,E). Such index values are observed for most resistant cultivars. However, some resistant cultivars differ significantly from the main group. The cultivar Syrah has high STI values for both biochemical parameters (STI = 1.0). The cultivar Bastardo has low index values for the biochemical parameters (0.01 and 0.012 for carotenoid and chlorophyll a + b contents, respectively) but high STI values for the number and length of second-order roots compared to other cultivars (0.96 and 0.4).

Sensitive cultivars are characterized by low tolerance index values for all phenotypic traits. The mean STI value is 0 for the number and length of second-order roots, 0.019 for the length of first-order roots and 0.089 for the number of new leaves. All sensitive cultivars demonstrate low rhizogenesis efficiency. Figure 3C shows the clustering of 30 grapevine cultivars into sensitive, intermediate and drought-resistant groups.

### 2.2. Phenotypic Response of Cultivars with Varying Levels of Resistance to Drought Stress

**Figure 4:**
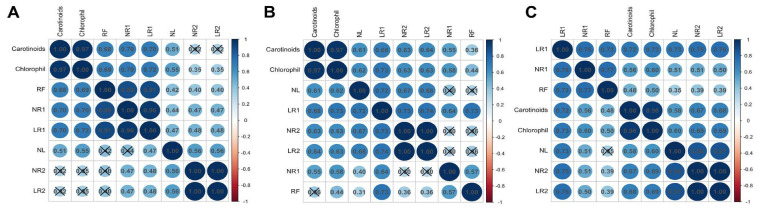
Spearman’s correlation coefficient between phenotypic traits in drought-sensitive genotypes (Saperavi) (**A**), genotypes with intermediate resistance (Kober 5BB) (**B**) and drought-resistant genotypes (Ferkal, Podarok Magaracha and Aligote) (**C**). The high and low intensity of color represent strong and weak relationships (blue for positive and red for negative) between the two variables, respectively. Values closer to one indicate a strong correlation, and a value closer to zero indicates a weaker relationship between the two variables. Crossed-out values indicate insignificant correlations between pair of parameters (significant level *p* < 0.01). The hierarchical clustering order was chosen for the correlation matrix. Spearman’s coefficients above the diagonal are given with Bonferroni correction for multiple comparisons.

Cultivars with different levels of tolerance to drought stress showed distinct responses to mannitol at various concentrations. The correlation matrices for phenotypic traits differ among the sensitive, intermediate and drought-resistant groups (see Figure 4). However, there are also similarities. For example, a strong positive correlation was observed for carotenoid and chlorophyll a + b contents (r = 0.97–1.0, *p* < 0.01), as well as between NR2 and LR2 (r = 0.97–1.0, *p* < 0.01), in all groups of cultivars. Other phenotypic traits showed a shift in the correlation matrix pattern in groups of cultivars with different levels of resistance. In the resistant group, the parameters NR2 and LR2 showed a strong positive correlation with the number of new leaves (NL) (r = 0.86 and r = 0.87, respectively, *p* < 0.01), as well as a positive correlation with LR1 (r = 0.75 and r = 0.76, respectively, *p* < 0.01). The correlation of second-order root characteristics with other phenotypic traits is weak (r < 0.7, *p* < 0.01) (Figure 4C). As drought resistance in plants decreases, the degree of correlation between NR2 and LR2 with other characteristics decreases; there is no correlation in sensitive cultivars (r = 0.35–0.56, *p* < 0.01) (Figure 4A). The opposite situation is observed for carotenoid and chlorophyll a + b contents: in resistant cultivars, a positive correlation is shown only with LR1 (r = 0.72 and r = 0.73 respectively, *p* < 0.01), which increases with other parameters as drought resistance decreases. Similarly, RF and NR1 in resistant cultivars show a positive correlation only with LR1 (r = 0.73–0.77, *p* < 0.01). In sensitive cultivars, all these parameters (carotenoid and chlorophyll a + b contents, RF, NR1 and LR1) form a cluster due to a strong positive correlation between them (r = 0.68–0.97, *p* < 0.01) (Figure 4A).

**Figure 5:**
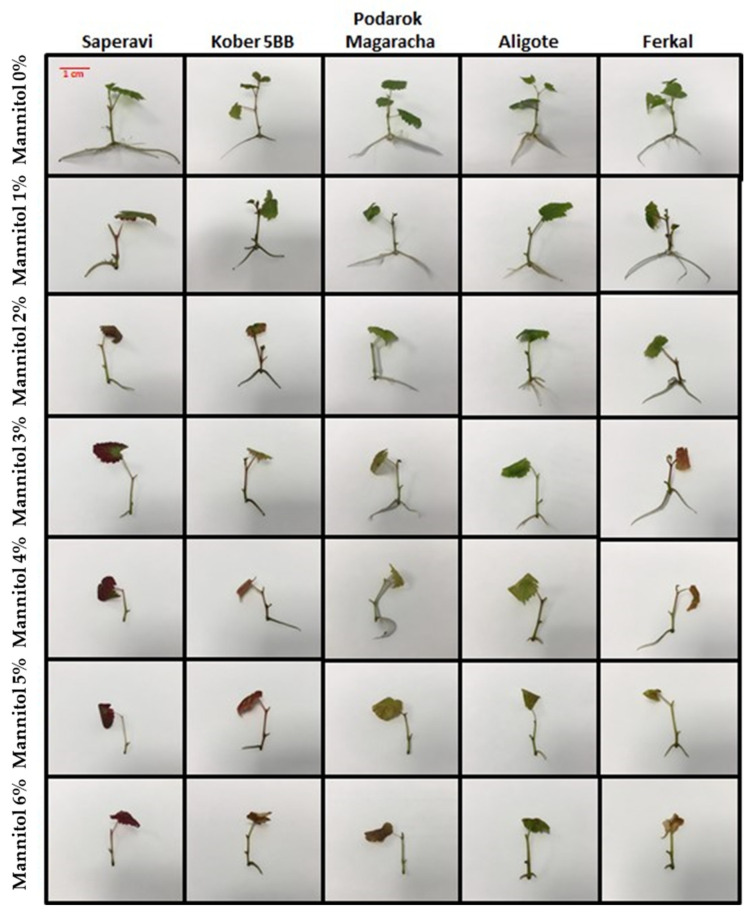
The effects of drought stress on the five genotypes [resistant (Ferkal, Podarok Magaracha and Aligote), intermediate (Kober 5BB) and sensitive (Saperavi)] when adding mannitol to the culture media at a concentration of 0 to 6% in 1% increments.

**Figure 6:**
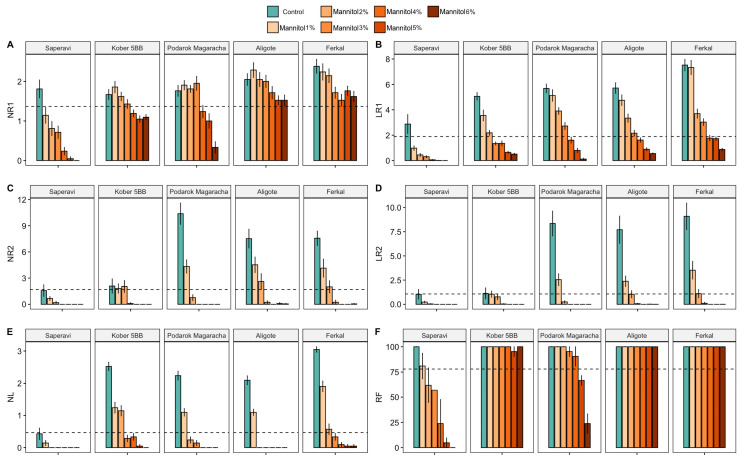
The statistics of the significant effects of drought stress on the study cultivars (Ferkal, Podarok Magaracha, Aligote, Kober 5BB and Saperavi), with the number of first-order roots (NR1) (**A**), the length of first-order roots (LR1) (**B**), the number of second-order roots (NR2) (**C**), the length of second-order roots (LR2) (**D**), the number of new leaves (NL) (**E**) and the risogenesis efficiency (RF) (**F**) under control and drought-stress conditions after 30 days. The vertical bars represent mean of 21 replicates ± SE. The dotted line indicates the mean of the parameter for all cultivars.

To further study the response of grapevines to drought, the following contrasting genotypes were selected for assessment: resistant (Ferkal, Podarok Magaracha and Aligote), intermediate (Kober 5BB) and sensitive (Saperavi). As resistant cultivars exhibit different tolerance indices for various phenotypic parameters, three such cultivars were selected. It was shown that drought stress significantly affected the shoot height, formation of new internodes and leaves, the number of roots and their length and functionality (Figure 5 and Figure 6).

Genotypes showed different responses. Under the influence of the stress factor, the height of shoots and the number and length of roots decreased sharply. Thus, in the sensitive Saperavi cultivar, after 30 days of anosis of leaves (except for the Aligote cultivar), a complete absence of second-order roots and a decrease in the length of formed first-order roots (significant in the Kober 5BB rootstock and almost complete in the Saperavi cultivar) were observed in all genotypes (Figure 5 and Figure 6B,C). Under the influence of the stress factor (mannitol), a significant or complete reduction in the growth processes of both the root and the shoot and partial or complete necrosis of the leaves in all the vines studied, regardless of the degree of resistance of the genotype, were observed (Figure 5). After cultivation on a medium with the addition of 1% mannitol, the complete inhibition of shoot growth and a decrease in the number and length of formed roots were observed. At the same time, the drought-resistant (Ferkal, Podarok Magarach, Aligote) and intermediate (Kober 5BB) genotypes did not differ from the control groups in terms of biometric parameters.

After 30 days of cultivation on a medium with the addition of 2% mannitol, the Saperavi cultivar showed the significant inhibition of rhizogenesis (an even greater reduction in the number and length of formed roots, a complete absence of second-order roots), partial necrosis and anthocyanosis of the leaves. In the Kober 5BB rootstock, after 30 days of cultivation on a medium with the addition of 2% mannitol, partial inhibition of rhizogenesis (a decrease in the number and length of formed roots) and partial anthocyanosis of the leaf blade were also noted. Drought-resistant (Ferkal, Podarok Magaracha, Aligote) genotypes demonstrated only the partial inhibition of shoot growth. After 30 days of cultivating explants on a medium with the addition of 3% mannitol, the inhibition of shoot growth, partial necrosis and anthocyanosis of leaves (except for the Aligote cultivar), a complete absence of second-order roots and a decrease in the length of formed first-order roots (significant in the Kober 5BB rootstock and almost complete in the Saperavi cultivar) were observed in all genotypes. A further increase in the content of mannitol in the culture medium led to a significant or complete reduction in the growth processes of both the root and the shoot and partial or complete necrosis of the leaves in all the vines studied, regardless of the degree of resistance of the genotype.

Taking the biometric and biochemical data for all 30 cultivars into account, a threshold value was calculated for each parameter, which is defined as the mean value for that parameter across all cultivars. Even under slight drought stress, all biometric parameters for the sensitive Saperavi cultivar typically fall below the mean values for all cultivars (Figure 6). Meanwhile, rhizogenesis efficiency remains high for cultivars with intermediate and high resistance. The number of new leaves (NL), the length of first-order roots (LR1), the number of second-order roots (NR2) and the length of second-order roots (LR2) all decrease with an increasing mannitol concentration in all cultivars. For all the resistant cultivars (Ferkal, Podarok Magaracha and Aligoté), the NR2 and LR2 values in the control group and at a 1% mannitol concentration are higher than the average values for these parameters across all the cultivars (Figure 6C,D). The Saperavi cultivar, which is sensitive, and the intermediate Kober 5BB rootstock have average biometric parameter values below the threshold, even in the control groups.

**Figure 7:**
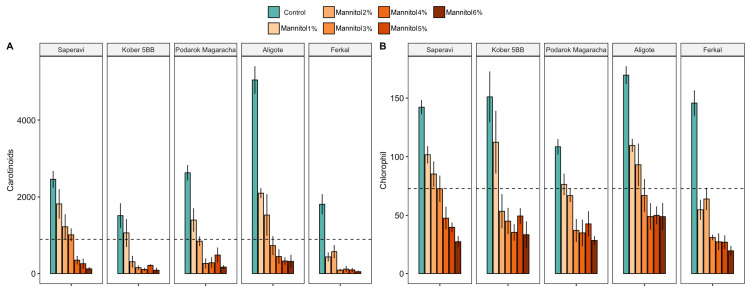
The statistics of the significant effects of drought stress on the study cultivars (Ferkal, Podarok Magaracha, Aligote, Kober 5BB and Saperavi), with the contents of carotenoids (**A**) and the contents of chlorophyll (a + b) (**B**) under control and drought-stress conditions after 30 days. The vertical bars represent the mean of 21 replicates ± SE. The dotted line indicates the mean of the parameter for all cultivars.

Biochemical indicators such as carotenoid and chlorophyll (a + b) contents change similarly across all cultivars, indicating stress in the plants (Figure 7). These parameters decrease as the mannitol concentration increases, crossing the threshold at 1% or 2%.

**Figure 8:**
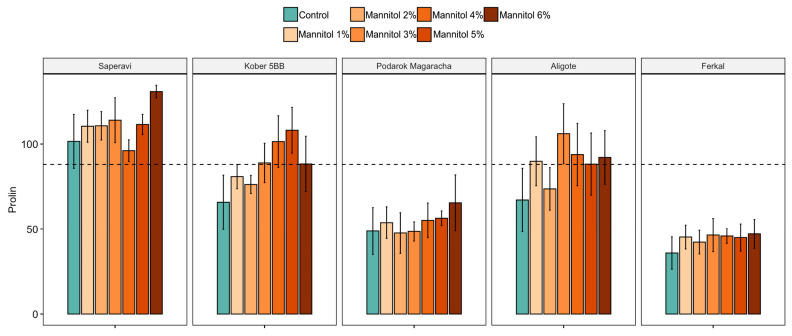
The statistics of the significant effects of drought stress on the study cultivars (Ferkal, Podarok Magaracha, Aligote, Kober 5BB and Saperavi), with the contents of proline under control and drought-stress conditions after 12 h. The vertical bars represent the mean of 21 replicates ± SE. The dotted line indicates the mean of the parameter for all cultivars.

The third biochemical parameter, the proline content, characterizes the rapid response to stress. Therefore, it was measured 12 h after the beginning of the stress period. For all cultivars, the proline content tended to increase as stress levels rose (Figure 8). The sensitive Saperavi cultivar exceeded the threshold even in the control group. Proline content values remained unchanged for the two resistant cultivars (Ferkal and Podarok Magaracha). The Kober 5BB rootstock and the Aligoté cultivar crossed the threshold as the concentration of mannitol increased. Overall, none of the cultivars showed significant changes after 12 h of drought stress. However, differences were observed between cultivars with varying degrees of resistance.

An analysis of all biometric and biochemical parameters showed that a 1% mannitol concentration is insignificant and does not exert a strong stress effect. Significant changes were observed in almost all parameters across all cultivars when the mannitol concentration was increased to 2% (Figure 6, Figure 7 and Figure 8).

### 2.3. Transcriptome Profile of Cultivars with Varying Levels of Resistance Exposed to Drought Stress

To reveal the molecular mechanism of the grapevine response to drought stress, 20 cDNA libraries of the above five grapevine genotypes with different resistance to control and drought stress were constructed and sequenced using the DNBSeq G400 platform (BGI); 712.11 million reads were produced via 150 nt paired-end sequencing from twenty libraries. The primary transcriptome sequencing data was deposited into the NCBI SRA archive (accession number SUB16149465). Quality control included the removal of adapters and low-quality reads. Raw reads were mapped to the reference sequences for major grapevine viruses and the human genome to eliminate potential contamination (the alignment rate was 0.66–3.55% for the human genome and was 0.13–3.10% for grapevine viruses with an average of 1.65% and 0.86%, respectively). No correlation was found between the number of reads for complementary viruses and transcriptional profiles. The cleaned dataset ranged from 23,926,502 to 44,695,307 reads with a 44% GC content.

In order to understand the common molecular mechanisms underlying tolerant cultivars, differentially expressed genes (DEGs) were identified in two groups of plants comprising all five cultivars: a control group and a group that was treated with 2% mannitol for 12 h. This approach identified similar changes in expression levels in response to drought stress, regardless of the plants’ degree of resistance.

**Figure 9:**
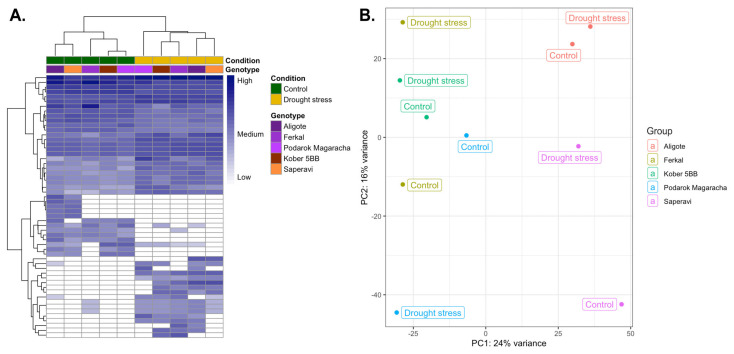
Transcriptome analysis of DEGs in five grapevine cultivars (Ferkal, Podarok Magaracha, Aligote, Kober 5BB and Saperavi) under drought stress. (**A**) Hierarchical cluster analysis of DEGs. Columns and rows in the heatmaps represent samples and genes, respectively. (**B**) Principal component analysis (PCA) of transcriptome data.

Only 55 DEGs were found for five libraries from the control group vs. five libraries from the stressed group, in which 33 genes were up-regulated and 22 were down-regulated. Cluster analysis of DEGs revealed a clear separation of plants into control and stress-exposed groups (Figure 9A). However, no clustering of cultivars according to their degree of drought tolerance was observed within each group.

A principal component analysis (PCA) was performed based on the gene expression DESeq level (Figure 9B). In the PCA scores plot, the first principal components (PCs) and second PCs accounted for 24% and 16% of the total variance among the two groups, respectively. Interestingly, principal component analysis of the differentially expressed genes did not reveal a clear distinction between the control and stressed plants. In this context, two types of cultivars can be identified based on PCA. For the Aligote cultivar and Kober 5BB rootstock, the control and stressed plants cluster quite closely together. However, for the Ferkal, Podarok Magaracha and Saperavi cultivars, the control and stressed samples differ significantly.

### 2.4. Functional Prediction and Pathway Enrichment Analysis of DEGs

**Figure 10:**
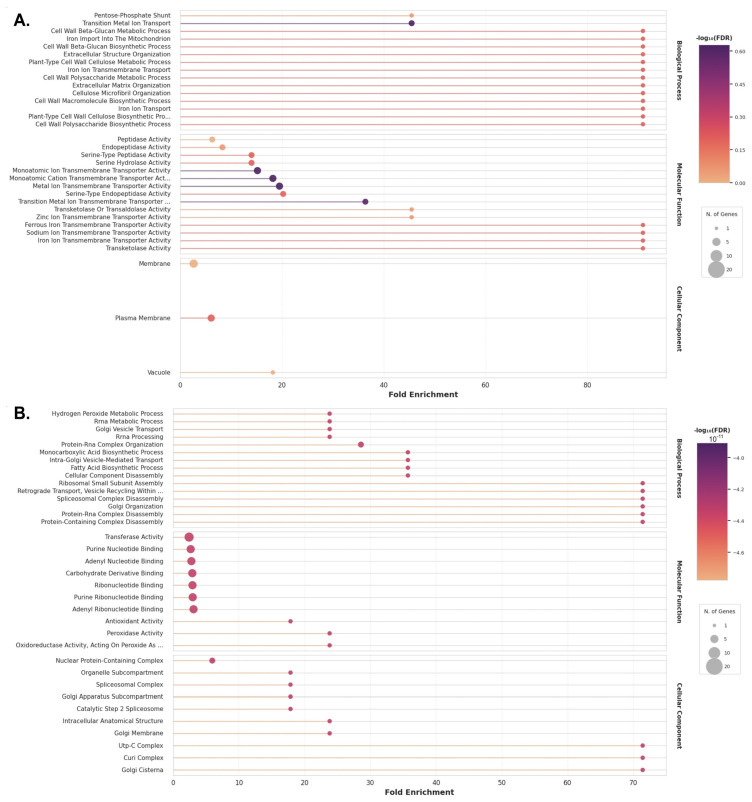
Gene Ontology (GO) enrichment analysis of DEGs across control and stressed groups of study cultivars (Ferkal, Podarok Magaracha, Aligote, Kober 5BB and Saperavi). The GO enrichment analysis highlights enriched terms with *p* < 0.05. The color intensity and size of each dot represent the −log10 (*p*-value) and the number of genes, respectively. (**A**) GO enrichment of up-regulated genes, (**B**) GO enrichment of down-regulated genes.

All DEGs were further subjected to Gene Ontology (GO) analysis. Matched DEGs were divided into three categories: biological processes, molecular functions and cellular components (Figure 10A,B). It should be noted that there is no overlap in GO terms between up-regulated and down-regulated DEGs across all three categories, and it is possible to identify biological processes that are activated and down-regulated in response to drought stress. The down-regulated DEGs in the biological process category were associated with transitional and transmembrane metal transport. It is likely that the beta-glucan, cellulose and polysaccharide metabolic processes are also inhibited, as well as the biosynthesis of macromolecules, cell-wall beta-glucan, cellulose and polysaccharide (Figure 10A). In contrast, the up-regulated DEGs in the biological process category were protein–RNA and protein-containing complex organization, intra-Golgi vesicle transport, hydrogen peroxide and rRNA metabolic process and biosynthesis of monocarboxylic and fatty acid (Figure 10B). The down-regulated and up-regulated DEGs according to the molecular function and cellular component also differ in response to drought stress (Figure 10A,B).

### 2.5. Identification of DEGs Between Cultivars with Different Drought Tolerance Under Drought Stress

**Figure 11:**
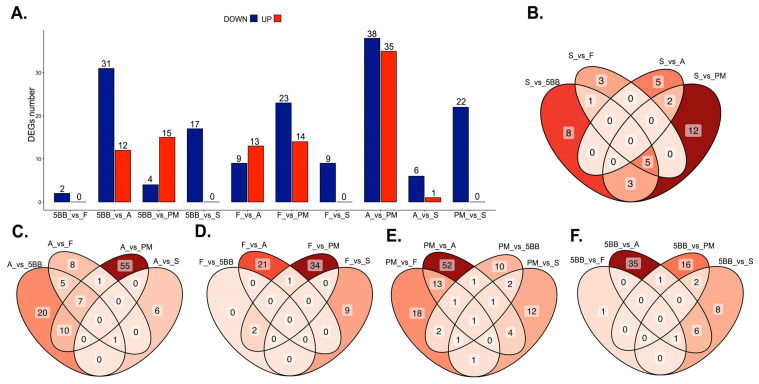
Differentially expressed genes (DEGs) between tested samples under stress conditions identified using RankProd with pfp < 0.05. (**A**) Numbers of DEGs compared between two samples are shown in red (up-regulated) and blue (down-regulated). (**B**) Venn diagram of the DEGs between Saperavi and other grapevine cultivars. (**C**) Venn diagram of the DEGs between Aligote and other grapevine cultivars. (**D**) Venn diagram of the DEGs between Ferkal and other grapevine cultivars. (**E**) Venn diagram of the DEGs between Podarok Magaracha and other grapevine cultivars. (**F**) Venn diagram of the DEGs between Kober 5BB rootstock and other grapevine cultivars. The DEG analyses was carried out for the comparison groups: 5BB_vs_F, 5BB_vs_A, 5BB_vs_PM, 5BB_vs_S, F_vs_A, F_vs_PM, F_vs_S, A_vs_PM, A_vs_S and PM_vs_S (where 5BB is Kober 5BB rootstock, F—Fercal, A—Aligote, PM—Podarok Magaracha and S—Saperavi).

To assess the differences in the way different grapevine cultivars respond to drought, according to their level of tolerance, additional libraries were sequenced after the plants had been exposed to 2% mannitol for six hours. A search was then conducted for DEGs between the cultivars. The DEG analyses was carried out for the next comparison groups: Kober 5BB_vs_Fercal, Kober 5BB_vs_Aligote, Kober 5BB_vs_Podarok Magaracha, Kober 5BB_vs_Saperavi, Fercal_vs_Aligote, Fercal_vs_Podarok Magaracha, Fercal_vs_Saperavi, Aligote_vs_Podarok Magaracha, Aligote_vs_Saperavi and Podarok Magaracha_vs_Saperavi. These analyses indicated that the expression patterns of DEG genes in all ten comparison groups were significantly different. The significantly up-regulated and down-regulated genes were identified with RankProd analyses (pfp < 0.05) (Figure 11A). When the DEGs of these comparison groups are represented by a Venn diagram, it was clear that both the unique and shared DEGs appeared between different pairs (Figure 11B–F).

The number of DEGs identified in the paired groups ranged from 2 to 73, with an average of 25. For some pairs of cultivars, the number of DEGs was low, indicating minor differences in gene expression patterns. For instance, only two DEGs were identified when comparing gene expression following exposure to 2% mannitol for the Kober 5BB_vs_Aligote group. In contrast, the Aligote_vs_Podarok Magaracha comparison group had the highest number of DEGs (73 in total) (Figure 11A).

When comparing the identified DEGs across paired groups under stress, practically no common DEGs were found, and many DEGs were unique to individual comparison groups (Figure 11B–F). For example, only 37 DEGs were identified for the Fercal_vs_Podarok Magaracha group; however, when identifying common DEGs in the Ferkal and other cultivars, 34 out of 37 were not identified in other comparison groups (Figure 11D).

No significantly up-regulated genes were detected in the Saperavi cultivar when it was compared with the more drought-tolerant plants (Figure 11A). However, genes that are more strongly expressed in the tolerant cultivars than in Saperavi were identified: 17 for Kober 5BB, 9 for Ferkal, 6 for Aligote and 22 for Podarok Magaracha. At the same time, the Venn diagram revealed no common genes for these groups (Figure 11B). Functional gene analyses and GO enrichment were performed for all of the identified genes. For the Fercal_vs_Saperavi group, genes involved in the following biological processes were more strongly expressed in the more resistant Ferkal rootstock: the ubiquitin-dependent protein catabolic process; the sulfur compound metabolic process; the modified amino acid metabolic process; and the glutathione metabolic process. For Kober 5BB_vs_Saperavi, the following were identified: modified amino acid metabolic process, glutathione metabolic process and methylation. For Aligote_vs_Saperavi, only methylation was found to be more expressed. For Podarok Magaracha_vs_Saperavi, the following processes were identified: modified amino acid metabolism, glutathione metabolism and methylation. Thus, although no common genes were identified for these comparison groups, genes involved in the same processes were more strongly expressed in the resistant cultivars under stress.

## 3. Discussion

One common approach is to compare contrasting genotypes and identify differentially expressed genes (DEGs) in stressed plants versus unstressed plants. However, the resistance mechanisms identified in model organisms cannot necessarily be applied to others [13]. For instance, a study of two contrasting soybean cultivars revealed that the expression of genes involved in calcium and MAPK signaling pathways is enhanced in the drought-tolerant cultivar. It was hypothesized that it responds to drought stress by regulating cell wall remodeling [41]. A study of contrasting wheat cultivars under drought conditions identified DEGs mainly involved in flavonoid biosynthesis, plant hormone, phenolamide and antioxidant pathways [42]. This indicates that different adaptive mechanisms have emerged in various groups of plants as a result of evolution.

In this study, we adopted a different approach involving the investigation of phenotypic changes and transcriptome profiles in several cultivars with varying degrees of drought stress resistance, using different concentrations of mannitol. Analyzing biometric and biochemical parameters enabled us to rank 30 *V. vinifera* cultivars according to their level of drought tolerance (Figure 3). Regardless of their tolerance level, all 30 genotypes exhibited certain common patterns in their response to stress. Thus, certain phenotypic traits decreased equally for all cultivars after a certain level of stress (see Figure 1 and Figure 2). For example, the length of primary roots (LR1) and carotenoid content decreased equally in all cultivars at mannitol concentrations of over 4%, while the number of leaves (NL) decreased at concentrations of over 3%. The most sensitive biometric characteristics were the number and length of second-order roots (NR2 and LR2), which decreased equally in all cultivars at concentrations of over 2% and were practically absent at concentrations of over 4%. Other parameters, such as the chlorophyll content, the number of first-order roots (NR1) and rhizogenesis efficiency (RF), showed significant variation among the cultivars, particularly under maximum stress conditions.

A shift in correlations among the parameters was observed for susceptible and resistant genotypes (Figure 4). For resistant cultivars, the correlation increases between the number of leaves and the number and length of second-order roots (NL, NR2 and LR2), while for sensitive cultivars, the correlation shifts towards rhizogenesis efficiency and the number and length of first-order roots (RF, NR1 and LR1). It should also be noted that, in the sensitive cultivar without stress exposure, the NL, NR2 and LR2 characteristics were below average for all cultivars (Figure 6), while the proline content was above average (Figure 8). This may indicate that sensitive cultivars have limited reserves for a compensatory response in the event of stress. Therefore, these phenotypic traits (NL, NR2 and LR2) can be used to evaluate not only the impact of stress on plants, but also the drought tolerance of grapevine cultivars.

An analysis of transcriptomic data from five genotypes with varying levels of resistance (Kober 5BB rootstock, cv. Aligoté, cv. Saperavi, cv. Podarok Magaracha and Ferkal rootstock) identified stress-induced differentially expressed genes in response to 2% mannitol. We had expected to identify patterns among cultivars with similar resistance levels, but none were found. Thus, cultivars that are quite similar based on phenotyping, such as Kober 5BB rootstock and cv. Aligote and cv. Podarok Magaracha (Figure 3), do not cluster into a single group based on either hierarchical cluster analysis of DEGs or a principal component analysis of transcriptome data (Figure 9). Furthermore, grape genotypes under normal conditions and those subjected to stress do not cluster into groups based on PCA either. These results are consistent with the variation in phenotypic characteristics across different cultivars (Figure 1 and Figure 2) and likely reflect the compensatory potential mentioned above.

Only a small number of common DEGs were identified for the five *V. vinifera* genotypes under drought stress. This could be explained by the fact that several cultivars with different responses to drought and levels of tolerance to this stress were examined. It can therefore be assumed that these cultivars do not share many of the same defense mechanisms. This view is supported by the observation of significant differences in transcriptomic profiles even among cultivars with similar tolerance levels (Figure 9B). Therefore, when comparing cultivars with different levels of drought tolerance, too few differentially expressed genes (DEGs) were found to be common to all of them. These genes probably reflect the general patterns of the grapevine response to this type of stress in all of the sensitive, intermediate and drought-tolerant cultivars (Figure 10). As in other studies [34,35,37,40,43], we have demonstrated that antioxidant activity is activated in all cultivars under drought conditions, regardless of their degree of drought tolerance. Along with the increase in antioxidant activity, genes associated with peroxidase and oxidoreductase activity are induced, indicating a cellular defense mechanism against excess reactive oxygen species (ROS) formed under stress. Shifts also occur in biosynthesis and metabolism. The biosynthesis of cell-wall beta-glucan, cellulose and polysaccharides is inhibited, while the biosynthesis of monocarboxylic and fatty acids increases. This was also observed in a study of the transcriptomic responses of the developing buds of the Merlot grapevine cultivar to drought-induced cell wall modification [40]. Another biological process that changes under drought stress in the five cultivars studied, which have varying levels of tolerance, is spliceosome complex disassembly. It has previously been suggested that alternative splicing plays a key post-transcriptional regulatory role in the response to single and combined stressors. In a study in which cv. Cabernet Sauvignon cuttings were subjected to drought stress, differentially expressed genes (DEGs) were enriched in the spliceosome pathway [38]. Another study investigated the leaves and roots of *V. vinifera* cv. Shine Muscat and cv. Thompson Seedless; it was found that the retained intron was the predominant type of differential alternative splicing event under drought stress [43]. Thus, all the biological processes listed above, which are affected during drought, have previously been reported in other studies, a finding that is supported by our data. Furthermore, for the first time, we have demonstrated that there is a decrease in the expression of genes associated with transitional and transmembrane metal transport, alongside an increase in intra-Golgi vesicle transport.

Thus, the main biological processes affected by drought in the five cultivars with different tolerance have been demonstrated, and these can likely be regarded as general patterns. Under drought conditions, the following were observed in all the varieties studied: (1) activation of antioxidant defense mechanisms; (2) a reduction in the biosynthesis of cell-wall beta-glucan, cellulose and polysaccharides, which is likely to lead to cell damage; (3) an increase in the biosynthesis of monocarboxylic and fatty acids; (4) an intensification of splicing processes; and (5) a reduction in transitional and transmembrane metal transport, accompanied by an increase in intra-Golgi vesicle transport.

It should be noted that the biological processes identified above do not address the specific characteristics of defense mechanisms in different cultivars, nor do they explain the principles of drought tolerance. To this end, we investigated differential gene expression in various cultivars under drought stress. Our results confirmed significant differences in the molecular defense mechanisms of cultivars with varying degrees of drought tolerance. These differences were observed even between cultivars with similar levels of tolerance, as evidenced by the varying numbers of DEGs identified, as well as the small number of genes shared according to the Venn diagram (Figure 11). Previous studies have demonstrated the different drought-resistance mechanisms in the two grapevine cultivars *V. vinifera* cv. Shine Muscat and *V. vinifera* cv. Thompson Seedless [43]. When comparing resistant and intermediate resistant cultivars with the susceptible Saperavi cultivar, it was shown that two processes are most frequently induced in resistant cultivars: glutathione metabolism and methylation.

Glutathione (GSH) is a small intracellular thiol molecule regarded as a potent non-enzymatic antioxidant. It acts as a substrate for both glutathione peroxidase and glutathione S-transferase, playing a role in the second phase of the detoxification of xenobiotics and cytotoxic molecules [44]. It is well established that glutathione and related antioxidants reduce osmotic stress in plants, the primary manifestation of drought [45]. The signaling function of GSH in *Arabidopsis thaliana* under drought stress conditions has also been demonstrated. The authors concluded that GSH transmits information about drought as perceived by the roots to the leaves during the early stages. This study compared the drought tolerance of wild-type plants with normal GSH expression levels with that of GSH-deficient mutants with reduced GSH expression levels. Wild-type plants demonstrated higher drought tolerance [46]. Another study investigated modified poplar plants with PtrGSTU23 overexpression and found that they had higher GST activity and lower reactive oxygen species (ROS) accumulation, resulting in increased drought tolerance [47].

DNA methylation is an epigenetic regulatory mechanism that dynamically interacts with plant responses to abiotic stresses, modulating gene expression and developmental processes. DNA methylation is known to play a significant role in drought stress in crops, including the dual regulation of gene expression via DNA methylation and the RNA-dependent DNA methylation pathway, as well as alternative splicing and long non-coding RNAs. While many studies have observed significant shifts in methylation levels across the genome or in gene promoters in drought-stressed plants, identifying the specific genes and pathways involved remains challenging [48,49,50]. For example, drought-induced hypermethylation was observed in the promoter of the cytokinin-oxidase gene in barley [51]. Studies of genomic differences in DNA methylation patterns between rice varieties with contrasting responses to drought and salinity revealed interplay among DNA methylation, gene expression and the small RNA content. These studies also suggest a more extensive role for DNA methylation in rice’s adaptation to abiotic stress [52]. Drought priming is a promising strategy for enhancing wheat’s tolerance to recurring drought. The results showed that light or moderate priming intensity had a positive effect on the drought-sensitive YM16 cultivar. Analysis revealed that the demethylation of TaP5CS and TaBADH, which are involved in the accumulation of osmolytes, contributes to the enhanced drought tolerance induced by priming [53].

Thus, these studies confirm our findings and support the hypothesis that increased expression of genes involved in glutathione metabolism and methylation may underpin the drought tolerance of grapevine cultivars.

## 4. Materials and Methods

### 4.1. Plant Materials and Growth Conditions

To curate a research collection of grapevines in vitro, vines of thirty genotypes [twenty six cultivars of all three ecogeographic groups: Aligote, Bastardo, Sauvignon, Syrah, Rkatsiteli, Cabernet Sauvignon, Malbec, Cabernet Franc, Pinot Blanc, Pinot Gris, Pinot Noir, Chardonnay, Riesling, Chasselas blanc and Garnacha blanca (*Vitis vinifera* convar. *occidentalis* Negr.); Muscat Blanc and Muscat Crima (*Vitis vinifera* convar. *orientalis* Negr.); Saperavi and Qoqur (*Vitis vinifera* convar. *pontica* Negr.); Akademik Avidzba, Veles, Kefesiya Magaracha, Yaltinskiy bessemyannyy, Livia, Podarok Magaracha and Ruta (*Vitis vinifera* × American *Vitis* hybrids); three rootstocks: Kober 5BB (*Vitis berlandieri* × *Vitis riparia*), Fercal (*Vitis berlandieri* × *Vitis vinifera*) and Selection Oppenheim 4 (SO4; *Vitis berlandieri* × *Vitis riparia*); one breeding form—Magarach no. TT2 (*V. vinifera* ‘Talisman’ × *V. vinifera* ‘Tomaisky’ selected by the All-Russian National Scientific Research Institute of Vine And Winemaking “Magarach”)] were collected from field-grown mother vines at the All-Russian National Scientific Research Institute of Vine And Winemaking “Magarach” (lat.: 44.850984′ N, long.: 33.650112′ E), vernalized for 1 month at a temperature of +4 °C and germinated in vessels with water. All vines were checked for cultivar suitability [54,55] both ampelographically and using the VIVC database with next microsatellite loci: VVS2 [56]; VVMD5 [57]; VVMD25, VVMD27 and VVMD28 [58]; VrZAG62 and VrZAG79 [59] (Table S1).

Further, in the greenhouse, growing green shoots were superficially sterilized [60]. The shoots were then cut into single-node cuttings and placed in tubes on modified MS (according to Zlenko et al. [61], described as PG medium, supplemented with 1% (*w*/*v*) sucrose and 0.75% (*w*/*v*) agar (Panreac, Spain)) and cultured at pH 5.7 and were maintained under a 16 h per day photoperiod with a tandem pair of fluorescent lamps, Philips (Amsterdam, The Netherlands) TL—D 36 W/54–765 and PHLUORAOSRAML 36 W/77 (light intensity of 65 µmol/m^2^s), at 25 ± 1 °C. After 2 months of cultivation on PG medium from single-node cuttings (without signs of bacterial or fungal infection), shoots developed, and they were cut off and placed in culture vessels (jar with a total volume of 500 mL) containing 50 mL agarized PG medium supplemented with 0.05 mg/L NAA, with seven plants per culture vessel [62].

### 4.2. Stress Treatments and Phenotyping

Stress treatments were carried out under in vitro conditions by adding the mannitol (D(-)-mannitol, PanReac, Barcelona, Spain) at concentrations ranging from 0.0 to 6.0% (*w*/*v*) in increments of 1.0% (drought) [63] to the PG medium. The osmotic pressure was calculated according to Lazareva et al. [64] and was as follows: 134.2 kPa for 1.0% mannitol, 268.5 kPa for 2.0% mannitol, 402.7 kPa for 3.0% mannitol, 536.95 kPa for 4.0% mannitol, 671.2 kPa for 5.0% mannitol and 805.4 kPa for 6.0% mannitol. Seven cuttings with two eyes were placed in each of the culture vessels, in three repetitions, for each variant of stress treatments.

After 6 and 12 h of cultivation, samples were taken to analyze the content of free proline in plant tissues according to the method described by Bates et al. [65]. The determination of free proline content was carried out with a ninhydrin-based protocol with some modifications [66,67]. Proline extraction was carried out by homogenizing a 100 mg leafy sample in 2 mL of 3% aqueous sulfosalicylic acid, and the homogenate was centrifuged (5 min at 8000× *g*), and after cooling and transferring 100 µL of supernatant to new tubes, a 0.5 mL ninhydrin reagent (1.25 g ninhydrin, 20 mL 6 M H_3_PO_4_, 30 mL glacial acetic acid) was added and incubated at 96C during 1 h. The reaction mixture was extracted with 1.0 mL of toluene and mixed vigorously for 15–20 s. The chromophore containing toluene was aspirated from the aqueous phase and warmed to room temperature, and the absorbance was read at 520 nm using toluene for a blank. The proline content (ng/100 mg fw) was determined from a calibration curve using proline (Serva, Heidelberg, Germany).

After 4 weeks of stress treatments, the biometric [risogenesis frequency (RF), number of new leaves (NL), number of first (NR1) and second (NR2) orders roots, length of first (LR1) and second (LR2) orders roots] and biochemical (contents of chlorophylls a, b and carotenoids in leaves) parameters of plants were measured [67] and calculated using the following formulas:

The risogenesis frequency (RF) was calculated as the quotient of the number of explants with developed roots (Nr) divided by the total number of explants (No); the results are expressed as a percentage: RF = (Nr/No) × 100;

The number of new leaves (NL) was calculated as the quotient of the number of developed new leaves on each explant (Nnl) divided by the total number of explants (No): NL = Nnl/No;

The number of first order roots (NR1) was calculated as the quotient of the number of developed first order roots on each explant (Nr1) divided by the total number of explants (No): NR1 = Nr1/No;

The number of second order roots (NR2) was calculated as the quotient of the number of developed second order roots on each explant (Nr2) divided by the total number of explants (No): NR2 = Nr2/No;

The length of first order roots (LR1) was calculated as the quotient of the length of developed first order roots on each explant (Nlr1) divided by the total number of explants (No): LR1 = Nlr1/No;

The length of second order roots (LR2) was calculated as the quotient of the length of developed second order roots on each explant (Nlr2) divided by the total number of explants (No): LR2 = Nlr2/No;

The photosynthetic pigment contents [chlorophylls a (Chla), b (Chlb) and carotenoids (Car)] were determined by extracting pigments from leaves with 96% ethyl alcohol [68]. The degree of solution absorption (optical density) for chlorophylls a, b, and carotenoids was determined using a spectrophotometer at a wavelength of 665, 649 and 471 nm, respectively.

Cchl a = 13.70D_665_ − 5.76 D_649_; Cchl b = 25.80 D_649_ − 7.60 D_665_; Ccar = (1000D_471_ × 2.13Cchl a − 97.64Cchl b)/209 A = C × V/1000 × n

where C is the pigment concentrations; D is the optical density; V is the extract volume; n is the leaf fresh weight; and A is the pigment content (ng/100 mg fw).

### 4.3. RNA Isolation, Library Preparation and Sequencing

RNA samples were extracted from plants (6 and 12 h from control and stress groups) using the Spectrum Plant Total RNA Kit (Sigma-Aldrich, Darmstadt, Germany). Each sample was treated with DNAse (Thermo Fisher Scientific, Waltham, MA, USA). The quantity and quality of total RNA were analyzed with a Qubit 4.0 (Thermo Fisher Scientific, USA) and stained with ethidium bromide in a 1.5% agarose gel. The cDNA was synthesized using Revert Aid Minus Reverse Transcriptase (Thermo Fisher Scientific, USA) according to the manufacturer’s protocol using oligo(dT) 18 primers. The libraries were synthesized with the NEBNext Ultra II DNA Library Prep Kit for Illumina (New England Biolabs, Ipswich, MA, USA), and paired-end sequencing as 150 nt reads was done on the BGI DNBSeq G400 instrument (MGI tech GmbH, Ltd., Berlin, Germany).

### 4.4. Bioinformatic Analyses

Quality and length trimming of the reads was conducted using Fastp v.0.20.0 [69]. To delete contamination reads, the mapping of reads to the reference human genome and grape viruses database was performed using Bowtie2 (Version 2.5.4) software [70]. The filtered reads for each sample were aligned with the reference genome *V. vinifera*, downloaded from NCBI (accession number GCF_030704535). The estimated counts were calculated using the pseudo-alignment to the reference genome method by Kallisto [71]. Differential expression gene (DEG) analysis was performed by using the R package DESeq2 [72,73]. Differential expression genes (DEGs) were identified based on the criterion (|log2Fold Change| ≥ 1 and FDR < 0.05). In the case of small samples, the RankProd approach was used to identify DEGs (FPF < 0.05) [74]. DESeq2 was chosen because it is stringent to detect outliers and excludes genes with extreme read counts by default and the false positive rate for DEGs is 0% at adjusted *p* values less than 0.05 [75,76].

For the functional annotation of differentially expressed genes in *V. vinifera*, we used the reference genome annotation IGGP 12X.v0 [77]. GO term enrichment analysis was performed using the GOATOOLS package v1.1.6 [78]. GO term enrichment was considered statistically significant at an FDR < 0.05 (Benjamini–Hochberg correction) and Fold Enrichment > 1.0. Results were categorized into three functional domains: biological process (BP), molecular function (MF) and cellular component (CC).

### 4.5. Statistical Analysis

Analysis of variance (ANOVA) was performed for all parameters to estimate the significance of the stress, cultivar and their interaction using the library “agricolae” in RStudio v.2024.12.1. The experiment design considered drought stress as the main factor and the cultivar as the subfactor. The method for adjusting *p* values used was Bonferroni. Means were separated using LSD at *p* ≤ 0.05. The cultivars were characterized for each phenotypic trait using the stress tolerance index (STI) [79]. The stress tolerance index (STI) was calculated for all thirty grapevine cultivars using the formula defined by Fernandez:

STI = Y_C_ × Y_S_/(X_C_)^2^

where Y_C_ and Y_S_ are the value of the phenotypic trait of a given cultivar under control and drought stress conditions, respectively; X_C_ is the mean of phenotypic trait of all cultivars under control and drought stress conditions, respectively.

The index was calculated for each phenotypic trait at each stress level (mannitol concentrations ranging from 1% to 6%). Then, the average index value was calculated for each phenotypic trait and cultivar. These values were then used for FANNY cluster analysis (bootstrap = 1000) and principal component analysis (PCA) performed using the library “factoextra” in R. The Spearman’s correlation analysis was performed using the libraries “psych” and “corrplot”. All graphs were constructed using the library “ggplot” in R.

## 5. Conclusions

Thus, an analysis of phenotypic characteristics was used to characterize the drought tolerance of 30 grape genotypes, enabling the study of the crop’s response to drought stress. The study revealed that the response of different grapevine genotypes to drought is linked to the activation of antioxidant defense systems, such as the induction of peroxidase and oxidoreductase. It is also associated with changes in the metabolism and biosynthesis of glucan, cellulose, polysaccharides, monocarboxylic acids and fatty acids, as well as metal transport. Meanwhile, more resilient cultivars exhibited enhanced glutathione metabolism and methylation processes, suggesting a role for epigenetics in adaptation. Despite exhibiting common responses, each cultivar employs unique molecular mechanisms, reflecting their evolutionary diversity. Transcriptomic data confirmed variability in defense strategies, even among cultivars with similar levels of resistance.

## Figures and Tables

**Table 1:** Response of phenotypic parameters to drought stress experienced by different grapevine cultivars.

Condition	NR1, pcs	LR1, cm	NR2, pcs	LR2, cm	NL, pcs	RF, %	Car.	Chlor.	Prol.
							ng/100 mg of Fresh Weight		
Control	2.14 ^a^	5.46 ^a^	5.97 ^a^	4.44 ^a^	1.97 ^a^	96.8 ^a^	2983.7 ^a^	159.0 ^a^	79.1 ^a^
Mannitol 1%	1.79 ^b^	3.01 ^b^	3.44 ^b^	2.04 ^b^	0.77 ^b^	90.3 ^ab^	1420.4 ^b^	106.1 ^b^	78.6 ^a^
Mannitol 2%	1.56 ^c^	1.96 ^c^	1.63 ^c^	0.68 ^c^	0.27 ^c^	88.9 ^ab^	767.9 ^c^	72.5 ^c^	83.7 ^a^
Mannitol 3%	1.34 ^d^	1.28 ^d^	0.58 ^d^	0.23 ^d^	0.17 ^c^	83.2 ^bc^	437.0 ^cd^	55.8 ^d^	92.5 ^a^
Mannitol 4%	1.15 ^e^	0.82 ^e^	0.15 ^d^	0.04 ^d^	0.05 ^d^	75.1 ^cd^	296.1 ^d^	45.0 ^de^	93.6 ^a^
Mannitol 5%	0.94 ^f^	0.52 ^ef^	0.06 ^d^	0.01 ^d^	0.02 ^d^	63.7 ^d^	198.9 ^d^	39.4 ^e^	94.0 ^a^
Mannitol 6%	0.65 ^g^	0.19 ^f^	0.02 ^d^	0.002 ^d^	0.01 ^d^	47.9 ^e^	144.0 ^d^	31.7 ^e^	94.8 ^a^

The mean values of morphometric and biochemical parameters were separated using the least significant difference (LSD) test at *p* ≤ 0.05, with the Bonferroni *p*-value adjustment method. Different letters in superscript indicate the significant treatment effect.

## Data Availability

The data that support the tables and figures in this study are available from the corresponding author upon reasonable request.

## Supplementary Materials

The following supporting information can be downloaded at: https://www.mdpi.com/article/10.3390/plants15101464/s1, Table S1: Analysis of grape genotypes based on seven microsatellite markers; Table S2: Stress tolerance indexes for grapevine cultivars.

## References

[1] Wang M., Vannozzi A., Wang G., Liang Y., Tornielli G., Zenoni S., Cavallini E., Pezzotti M., Cheng Z. (2014). Genome and transcriptome analysis of the grapevine (*Vitis vinifera* L.) WRKY gene family. Hortic. Res..

[2] Bonarota M.-S., Toups H.S., Bristow S.T., Santos P., Jackson L.E., Cramer G.R., Barrios-Masias F.H. (2024). Drought response and recovery mechanisms of grapevine rootstocks grafted to a common *Vitis vinifera* scion. Plant Stress.

[3] Król A., Weidner S. (2017). Changes in the proteome of grapevine leaves (*Vitis vinifera* L.) during long-term drought stress. J. Plant Physiol..

[4] Mazzucato M., Okonjo-Iweala N., Rockström J., Shanmugaratnam T. (2023). Turning the tide: A call to collective action. Glob. Comm. Econ. Water.

[5] Charrier G., Delzon S., Domec J.C., Zhang L., Delmas C.E., Merlin I., Corso D., King A., Ojeda H., Ollat N. (2018). Drought will not leave your glass empty: Low risk of hydraulic failure revealed by long-term drought observations in world’s top wine regions. Sci. Adv..

[6] Gambetta G.A., Herrera J.C., Dayer S., Feng Q., Hochberg U., Castellarin S.D. (2020). The physiology of drought stress in grapevine: Towards an integrative definition of drought tolerance. J. Exp. Bot..

[7] Naulleau A., Gary C., Prévot L., Hossard L. (2021). Evaluating strategies for adaptation to climate change in grapevine production—A systematic review. Front. Plant Sci..

[8] Serra I., Strever A., Myburgh P.A., Deloire A. (2014). The interaction between rootstocks and cultivars (*Vitis vinifera* L.) to enhance drought tolerance in grapevine. Aust. J. Grape Wine Res..

[9] Knipfer T., Eustis A., Brodersen C., Walker A.M., McElrone A.J. (2015). Grapevine species from varied native habitats exhibit differences in embolism formation/repair associated with leaf gas exchange and root pressure. Plant Cell Environ..

[10] Zhang L., Marguerit E., Rossdeutsch L., Ollat N., Gambetta G.A. (2016). The influence of grapevine rootstocks on scion growth and drought resistance. Theor. Exp. Plant Physiol..

[11] Yıldırım K., Yağcı A., Sucu S., Tunç S. (2018). Responses of grapevine rootstocks to drought through altered root system architecture and root transcriptomic regulations. Plant Physiol. Biochem..

[12] Barrios-Masias F.H., Knipfer T., Walker M.A., McElrone A.J. (2018). Differences in hydraulic traits of grapevine rootstocks are not conferred to a common *Vitis vinifera* scion. Funct. Plant Biol..

[13] Cochetel N., Ghan R., Toups H.S., Degu A., Tillett R.L., Schlauch K.A., Cramer G.R. (2020). Drought tolerance of the grapevine, *Vitis champinii* cv. Ramsey, is associated with higher photosynthesis and greater transcriptomic responsiveness of abscisic acid biosynthesis and signaling. BMC Plant Biol..

[14] Cuneo I.F., Barrios-Masias F.H., Knipfer T., Uretsky J., Reyes C., Lenain P., Brodersen C.R., Walker M.A., McElrone A.J. (2021). Differences in grapevine rootstock sensitivity and recovery from drought are linked to fine root cortical lacunae and root tip function. New Phytol..

[15] Liang W., Wang X., Wang H., Yan A., Ren I., Liu Z., Sun L. (2026). Advancements in Genetic Transformation of Grapevine (*Vitis* spp.). Horticulturae.

[16] Sahu M., Maurya S., Jha Z. (2023). In vitro selection for drought and salt stress tolerance in rice: An overview. Plant Physiol. Rep..

[17] Suzuki N., Miller G., Morales J., Shulaev V., Torres M.A., Mittler R. (2011). Respiratory burst oxidases: The engines of ROS signaling. Curr. Opin. Plant Biol..

[18] Turkan I., Demiral T. (2009). Recent developments in understanding salinity tolerance. Environ. Exp. Bot..

[19] Munns R., Tester M. (2008). Mechanisms of salinity tolerance. Annu. Rev. Plant Biol..

[20] Hochberg U., Degu A., Toubiana D., Gendler T., Nikoloski Z., Rachmilevitch S., Fait A. (2013). Metabolite profiling and network analysis reveal coordinated changes in grapevine water stress response. BMC Plant Biol..

[21] Ashraf M., Foolad M.R. (2007). Roles of glycine betaine and proline in improving plant abiotic stress resistance. Environ. Exp. Bot..

[22] Rai M.K., Jaiswal V.S., Jaiswal U. (2010). Regeneration of plantlets of guava (*Psidium guajava* L.) from somatic embryos developed under salt-stress condition. Acta Physiol. Plant..

[23] Kishore G.K., Pande S., Podile A.R. (2005). Biological control of late leaf spot of peanut (*Arachis hypogaea*) with chitinolytic bacteria. Phytopathology.

[24] Comas L., Becker S., Cruz V.M.V., Byrne P.F., Dierig D.A. (2013). Root traits contributing to plant productivity under drought. Front. Plant Sci..

[25] Abdelraheem A., Esmaeili N., O’Connell M., Zhang J. (2019). Progress and perspective on drought and salt stress tolerance in cotton. Ind. Crops Prod..

[26] Thompson M.R., Douglas T.J., Obata-Sasamoto H., Thorpe T.A. (1986). Mannitol metabolism in cultured plant cells. Physiol. Plant..

[27] Hohl M., Schopfer P. (1991). Water relations of growing maize coleoptiles: Comparison between mannitol and Polyethylene Glycol 6000 as external osmotica for adjusting turgor pressure. Plant Physiol..

[28] Osmolovskaya N., Shumilina J., Kim A., Didio A., Grishina T., Bilova T., Keltsieva O.A., Zhukov V., Tikhonovich I., Tarakhovskaya E. (2018). Methodology of drought stress research: Experimental setup and physiological characterization. Int. J. Mol. Sci..

[29] Munns R. (2002). Comparative physiology of salt and water stress. Plant Cell Environ..

[30] Zang X., Komatsu S. (2007). A proteomics approach for identifying osmotic-stress-related proteins in rice. Phytochem.

[31] Chutipaijit S. (2016). Changes in physiological and antioxidant activity of indica rice seedlings in response to mannitol-induced osmotic stress. Chil. J. Agric. Res..

[32] Ali M.N., Yeasmin L., Gantait S., Goswami R., Chakraborty S. (2014). Screening of rice landraces for salinity tolerance at seedling stage through morphological and molecular markers. Physiol. Mol. Biol. Plants.

[33] Agarwal P., Agarwal P.K., Sopory S.K. (2006). Role of DREB transcription factors in abiotic and biotic stress tolerance in plants. Plant Cell Rep..

[34] Zhang X., Zheng Q., Hao Y., Zhang Y., Gu W., Deng Z., Zhou P., Fang Y., Chen K., Zhang K. (2025). Physiology and transcriptome profiling reveal the drought tolerance of five grape varieties under high temperatures. J. Integr. Agric..

[35] Özmen C.Y., Baydu F.Y., Ergül A. (2025). Comparative analysis of Cabernet Sauvignon (*Vitis vinifera* L.) and Kober 5BB (*V. berlandieri* × *V. riparia*) root transcriptomes reveals multiple processes associated with drought tolerance in grapevines. Horticulturae.

[36] Haider M.S., Kurjogi M.M., Khalil-Ur-Rehman M., Fiaz M., Pervaiz T., Jiu S., Haifeng J., Chen W., Fang J. (2017). Grapevine immune signaling network in response to drought stress as revealed by transcriptomic analysis. Plant Physiol. Biochem..

[37] Haider M.S., Zhang C., Kurjogi M.M., Pervaiz T., Zheng T., Zhang C., Lide C., Shangguan L., Fang J. (2017). Insights into grapevine defense response against drought as revealed by biochemical, physiological and RNA-Seq analysis. Sci. Rep..

[38] Ju Y.L., Min Z., Zhang Y., Zhang K.K., Liu M., Fang Y.L. (2021). Transcriptome profiling provide new insights into the molecular mechanism of grapevine response to heat, drought, and combined stress. Sci. Hortic..

[39] Ma W., Lu S., Li W., Nai G., Ma Z., Li Y., Chen B., Mao J. (2023). Transcriptome and metabolites analysis of water-stressed grape berries at different growth stages. Physiol. Plant..

[40] Girardi F., Canton M., Bettio G., Rasori A., Cardillo V., Meggio F., Botton A. (2026). Physiological responses of grapevine (*Vitis vinifera* L.) developing buds to drought stress: A transcriptomic analysis. OENO One.

[41] Xuan H., Huang Y., Zhou L., Deng S., Wang C., Xu J., Wang H., Zhao J., Guo N., Xing H. (2022). Key soybean seedlings drought-responsive genes and pathways revealed by comparative transcriptome analyses of two cultivars. Int. J. Mol. Sci..

[42] Niu Y., Li J., Sun F., Song T., Han B., Liu Z., Su P. (2023). Comparative transcriptome analysis reveals the key genes and pathways involved in drought stress response of two wheat (*Triticum aestivum* L) varieties. Genomics.

[43] Lin Y., Liu S., Fang X., Ren Y., You Z., Xia J., Hakeem A., Yang Y., Wang L., Fang J. (2023). The physiology of drought stress in two grapevine cultiFvars: Photosynthesis, antioxidant system, and osmotic regulation responses. Physiol. Plant..

[44] Hasanuzzaman M., Nahar K., Anee T.I., Fujita M. (2017). Glutathione in plants: Biosynthesis and physiological role in environmental stress tolerance. Physiol. Mol. Biol. Plants.

[45] Kocsy G., Szalai G., Galiba G. (2002). Induction of glutathione synthesis and glutathione reductase activity by abiotic stresses in maize and wheat. Sci. World J..

[46] Koffler B.E., Luschin-Ebengreuth N., Stabentheiner E., Müller M., Zechmann B. (2014). Compartment specific response of antioxidants to drought stress in *Arabidopsis*. Plant Sci..

[47] Niu M.X., Feng C.H., He F., Zhang H., Bao Y., Liu S.J., Liu X., Su Y., Liu C., Wang H.L. (2024). The miR6445-NAC029 module regulates drought tolerance by regulating the expression of glutathione S-transferase U23 and reactive oxygen species scavenging in *Populus*. New Phytol..

[48] Rao X., Yang S., Lü S., Yang P. (2024). DNA methylation dynamics in response to drought stress in crops. Plants.

[49] Fan Y., Sun C., Yan K., Li P., Hein I., Gilroy E.M., Kear P., Bi Z., Yao P., Liu Z. (2024). Recent advances in studies of genomic DNA methylation and its involvement in regulating drought stress response in crops. Plants.

[50] Yadav S., Meena S., Kalwan G., Jain P.K. (2024). DNA methylation: An emerging paradigm of gene regulation under drought stress in plants. Mol. Biol. Rep..

[51] Surdonja K., Eggert K., Hajirezaei M.R., Harshavardhan V.T., Seiler C., Von Wirén N., Sreenivasulu N., Kuhlmann M. (2017). Increase of DNA methylation at the HvCKX2. 1 promoter by terminal drought stress in barley. Epigenomes.

[52] Garg R., Narayana Chevala V.V.S., Shankar R., Jain M. (2015). Divergent DNA methylation patterns associated with gene expression in rice cultivars with contrasting drought and salinity stress response. Sci. Rep..

[53] Li Q., Wang X., Sun Z., Wu Y., Malkodslo M.M., Ge J., Jing Z., Zhou Q., Cai J., Zhong Y. (2023). DNA methylation levels of TaP5CS and TaBADH are associated with enhanced tolerance to PEG-induced drought stress triggered by drought priming in wheat. Plant Physiol. Biochem..

[54] This P., Jung A., Boccacci P., Borrego J., Botta R., Costantini L., Crespan M., Dangl G.S., Eisenheld C., Ferreira-Monteiro F. (2004). Development of a standard set of microsatellite reference alleles for identification of grape cultivars. Theor. Appl. Genet..

[55] Cretazzo E., Moreno Sanz P., Lorenzi S., Benítez M.L., Velasco L., Emanuelli F. (2022). Genetic Characterization by SSR Markers of a Comprehensive Wine Grape Collection Conserved at Rancho de la Merced (Andalusia, Spain). Plants.

[56] Thomas M.R., Scott N.S. (1993). Microsatellite repeats in grapevine reveal DNA polymorphisms when analysed as sequence-tagged sites (STSs). Theor. Appl. Genet..

[57] Bowers J.E., Dangl G.S., Vignani R., Meredith C.P. (1996). Isolation and characterization of new polymorphic simple sequence repeat loci in grape (*Vitis vinifera* L.). Genome.

[58] Bowers J.E., Dangl G.S., Meredith C.P. (1999). Development and characterization of additional microsatellite DNA markers for grape. Am. J. Enol. Vitic..

[59] Sefc K.M., Regner F., Turetschek E., Glössl J., Steinkellner H. (1999). Identification of microsatellite sequences in *Vitis riparia* and their applicability for genotyping of different *Vitis* species. Genome.

[60] Maletich G., Pushin A., Rybalkin E., Plugatar Y., Dolgov S., Khvatkov P. (2024). Organogenesis in a Broad Spectrum of Grape Genotypes and Agrobacterium-Mediated Transformation of the Podarok Magaracha Grapevine Cultivar. Plants.

[61] Zlenko V.A., Likhovskoy V.V., Volynkin V.A., Khvatkov P.A., Vasilyk I.A., Dolgov S.V. (2017). Induction of in vitro somatic embryogenesis grapes (*Vitis vinifera* L.) of domestic and foreign breeding. Biotechnologiya.

[62] Maletich G., Gavrilenko I., Pushin A., Chelombit S., Khmelnitskaya T., Plugatar Y., Dolgov S., Khvatkov P. (2025). Somatic embryogenesis and *Agrobacterium*-mediated transformation in a number of grape cultivars. Plant Cell Tiss. Org..

[63] Tarchoun N., Saadaoui W., Mezghani N., Pavli O.I., Falleh H., Petropoulos S.A. (2022). The effects of salt stress on germination, seedling growth and biochemical responses of Tunisian squash (*Cucurbita maxima* Duchesne) germplasm. Plants.

[64] Lazareva E.M., Baranova E.N., Smirnova E.A. (2017). Reorganization of interphase microtubules in root cells of *Medicago sativa* L. during acclimation to osmotic and salt stress. Cell Tiss. Biol..

[65] Bates L.S., Waldren R.P., Teare I.D. (1973). Rapid determination of free proline for water-stress studies. Plant Soil.

[66] Raldugina G.N., Evsukov S.V., Bogoutdinova L.R., Gulevich A.A., Baranova E.N. (2021). Morpho-physiological testing of NaCl sensitivity of tobacco plants overexpressing choline oxidase gene. Plants.

[67] Khaliluev M.R., Bogoutdinova L.R., Raldugina G.N., Baranova E.N. (2022). A simple and effective bioassay method suitable to comparative in vitro study of tomato salt tolerance at early development stages. Methods Protoc..

[68] Shlyk A.A. (1971). Definition of a Chlorophyll and Carotenoids in Extracts of Green Leaves. Biochemical Methods in Physiology of Plants.

[69] Chen S., Zhou Y., Chen Y., Gu J. (2018). Fastp: An ultra-fast all-in-one FASTQ preprocessor. Bioinformatics.

[70] Langmead B., Salzberg S.L. (2012). Fast gapped-read alignment with bowtie 2. Nat. Methods.

[71] Bray N.L., Pimentel H., Melsted P., Pachter L. (2016). Near-optimal probabilistic RNA- seq quantification. Nat. Biotechnol..

[72] Love M.I., Huber W., Andes S. (2014). Moderated estimation of fold change and dispersion for RNA-seq data with DESeq2. Genome Biol..

[73] Varet H., Brillet-Guéguen L., Coppée J.Y., Dillies M.A. (2016). SARTools: A DESeq2- and EdgeR-based R pipeline for comprehensive differential analysis of RNA-Seq data. PLoS ONE.

[74] Hong F., Breitling R., McEntee C.W., Wittner B.S., Nemhauser J.L., Chory J. (2006). RankProd: A bioconductor package for detecting differentially expressed genes in meta-analysis. Bioinformatics.

[75] Anders S., McCarthy D.J., Chen Y., Okoniewski M., Smyth G.K., Huber W., Robinson M.D. (2013). Count-based differential expression analysis of RNA sequencing data using R and Bioconductor. Nat. Protoc..

[76] Rajkumar A.P., Qvist P., Lazarus R., Lescai F., Ju J., Nyegaard M., Mors O., Børglum A.D., Li Q., Christensen J.H. (2015). Experimental validation of methods for differential gene expression analysis and sample pooling in RNA-seq. BMC Genom..

[77] Flutre T. (2015). BSgenome.Vvinifera.URGI.IGGP12Xv0: Full Reference Nuclear Genome Sequences for Vitis Vinifera subsp. Vinifera PN40024 (IGGP version 12Xv0), R Package Version 2015. https://bioconductor.org/packages/release/data/annotation/html/BSgenome.Vvinifera.URGI.IGGP12Xv2.html.

[78] Klopfenstein D.V., Zhang L., Pedersen B.S., Ramírez F., Vesztrocy A.W., Naldi A., Mungall C.J., Yunes J.M., Botvinnik O., Weigel M. (2018). GOATOOLS: A Python library for Gene Ontology analyses. Sci. Rep..

[79] Fernandez G.C.J. (1992). Stress tolerance index—A new indicator of tolerance. HortScience.

