# Supplementary material for: Comparative Phenotype and Transcriptome Profiling in Some Grapevine Cultivars in Response to Drought Stress
Source: Plants (Basel). 2026 May 11;15(10):1464. doi: 10.3390/plants15101464 (PMC13210752; doi:10.3390/plants15101464)
Supplement: Supplementary file 1 [file plants-15-01464-s001.zip › plants-4266278-supplementary/Table S1.pdf]

Table S1. Analysis of grape genotypes based on seven microsatellite markers

Conformity to cultivar according to ampelographic description	VVS2		VVMD5		VVMD25		VVMD27		VVMD28		VrZAG62		VrZAG79		Matching the variety according to the VIVC database
Rkatsiteli	133	141	236	242	239	267	180	182	236	244	190	200	249	259	RKATSITELI
Akademik Avidzba	135	149	228	238	249	255	186	186	236	258	186	188	251	255	Genotype not found in VIVC database
Veles	133	141	236	238	241	267	182	182	236	236	190	190	253	259	Genotype not found in VIVC database
Kefesiya Magaracha	133	133	234	240	241	241	182	182	236	260	188	188	247	251	Genotype not found in VIVC database
Yaltinskiy bessemyannyy	143	153	230	236	245	255	182	182	244	246	196	202	247	247	Genotype not found in VIVC database
Livia	135	149	236	240	253	253	190	195	244	244	188	188	255	259	Genotype not found in VIVC database
Muscat Crima	133	141	230	240	241	249	190	194	218	244	188	198	243	255, 259	Genotype not found in VIVC database
Podarok Magaracha	139	149	236	266	243	255	186	190	238	238	188	188	259	259	PODAROK MAGARACHA
Ruta	135	145	238	240	239	255	186	194	218	258	188	194	251	259	Genotype not found in VIVC database
Qoqur	145	145	230	240	239	255	188	188	258	258	194	204	251	251	KOKUR BELYI
Aligote	133	137	230	242	239	239	180	190	228	236	194	196	243	245	ALIGOTE
Bastardo	143	151	240	240	249	255	176	190	234	248	188	188	245	247	BASTARDO BLANCO, BASTARDO ROXO, TROUSSEAU GRIS, TROUSSEAU NOIR
Sauvignon	133	151	230	234	241	249	176	190	234	236	188	194	245	247	SAUVIGNON
Syrah	133	133	228	234	241	241	190	192	218	228	188	194	245	251	SYRAH

Cabernet Sauvignon	139	151	234	242	239	249	176	190	234	236	188	194	247	247	CABERNET SAUVIGNON
Malbec	133	151	230	240	239	249	190	192	234	268	188	202	245	259	Genotype not found in VIVC database
Cabernet franc	139	147	228	242	239	255	182	190	228	236	194	204	247	259	CABERNET FRANC
Muscat Blanc	133	133	230	238	241	249	180	195	246	268	186	196	251	255	MUSCAT A PETITS GRAINS BLANCS, MUSCAT A PETITS GRAINS NOIRS, MUSCAT A PETITS GRAINS ROSES, MUSCAT A PETITS GRAINS ROUGES
Pinot Blanc	137	151	230	240	239	249	186	190	218	236	188	194	239	245	PINOT BLANC, PINOT GRI, PINOT NOIR
Pinot Gris	137	151	230	240	239	249	186	190	218	236	188	194	239	245	PINOT BLANC, PINOT GRI, PINOT NOIR
Pinot Noir	137	151	230	240	239	249	186	190	218	236	188	194	239	245	PINOT BLANC, PINOT GRI, PINOT NOIR
Chardonnay	137	143	236	240	239	255	182	190	218	228	188	196	243	245	CHARDONNAY
Riesling	143	151	228	236	249	255	182	190	228	234	194	204	243	245	RIESLING ROT, RIESLING WEISS
Saperavi	133	145	226	242	239	241	190	193	235	244	188	200	243	261	SAPERAVI
Chasselas blanc	133	143	230	237	251	255	186	190	218	268	194	204	251	259	CHASSELAS BLANC
Garnacha blanca	149	149	230	234	249	249	180	194	226	268	186	188	259	261	Genotype not found in VIVC database
Magarach no. TT2	133	141	236	238	241	267	182	182	236	236	190	190	253	259	Genotype not found in VIVC database
Kober 5BB	147	147	228	228	239	251	192	208	236	244	192	214	251	255	Genotype not found in VIVC database
Fercal	143	143	238	266	237	255	184	190	220	244	184	220	245	257	FERCAL
Selection Oppenheim 4	151	151	238	238	239	251	202	210	216	236	200	214	251	255	Genotype not found in VIVC database
